# The big challenges in modeling human and environmental well-being

**DOI:** 10.12688/f1000research.7636.1

**Published:** 2016-04-13

**Authors:** Shripad Tuljapurkar

**Affiliations:** 1 Department of Biological Sciences, Stanford University, Stanford, California, USA

**Keywords:** sustainable development, modeling, human and environmental well-being, population, inequality

## Abstract

This article is a selective review of quantitative research, historical and prospective, that is needed to inform sustainable development policy. I start with a simple framework to highlight how demography and productivity shape human well-being. I use that to discuss three sets of issues and corresponding challenges to modeling: first, population prehistory and early human development and their implications for the future; second, the multiple distinct dimensions of human and environmental well-being and the meaning of sustainability; and, third, inequality as a phenomenon triggered by development and models to examine changing inequality and its consequences. I conclude with a few words about other important factors: political, institutional, and cultural.

## Introduction

**Figure 1.**
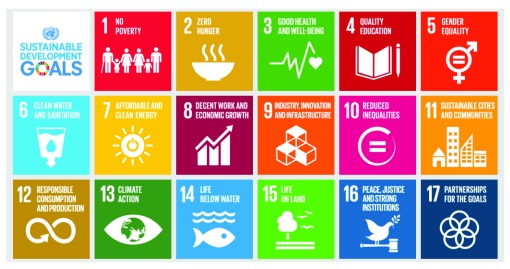
Sustainable development goals. Adopted by the United Nations in 2015. Available with much other material at https://sustainabledevelopment.un.org/.

The latest United Nations (UN) forecast says that the world population will likely increase from about 7 billion today to about 10.5 billion in 2100 ^1^. A UN-supported analysis of global well-being ^2^ highlights the costs of development “to ecosystem health, biodiversity, air quality, and climate resiliency”. These trends have motivated a vigorous and growing body of research, policy, opinion, and discussion, much of it polarized, emphasizing either the environment or people. But there is growing acknowledgment that “environmental health and human health are fundamentally linked” ( 3, an example from the ecological literature). That linkage goes beyond ‘health’ to encompass many dimensions that constitute the ‘well-being’ of humans and the environment. Many of these dimensions are evident in the UN’s new sustainable development goals, or SDGs ^4^, illustrated in Figure 1.

The concepts of development, sustainability, human well-being, and environmental well-being are complex. The term ‘development’ is usually defined in economic terms but (as explained later) has been extended to include some environmental attributes. ‘Sustainability’ is a widely used but vague term that can be operationalized in different and not always consistent ways (as indicated later).

**Figure 2.**
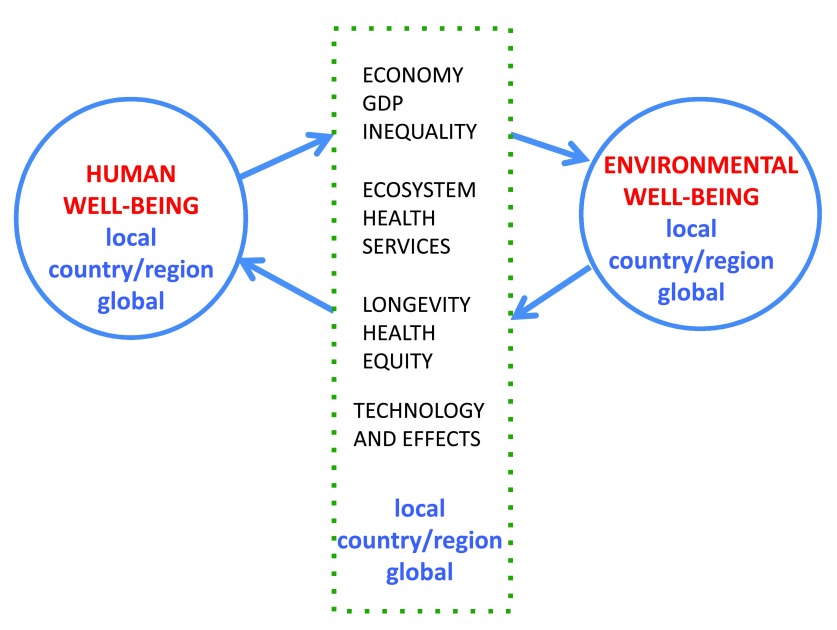
Feedbacks between human well-being and environmental well-being. The central box lists key elements of the two, many of which interact. Most of these dimensions affect both humans and environment. GDP, gross domestic product.

Human well-being ( Figure 2) includes obvious factors such as individual and population health but also other conditions of life (e.g. the freedoms discussed by Sen ^5^). Environmental well-being ( Figure 2) includes well-known aspects (e.g. viable and diverse ecosystems) but also less obvious factors (e.g. the regional effects of technology transfer). An important but often neglected aspect of both kinds of well-being is their distribution within and between countries. As Pope Francis ^6^ put it, the improvement of human well-being requires that we “protect the vulnerable in our world and … stimulate integral and inclusive models of development”. Much the same can be said of ecosystems. Human and environmental well-being feed back on each other (as in Figure 2) via many pathways (demographic, economic, biological, individual, and institutional). These feedbacks (some illustrated in Figure 2) span a range of scales in space (local, regional, country, and global) and time (short: years to decades; medium: several human generations; long: millennia). These feedbacks are also complex enough that substantive research usually focuses on just a few.

This invited article uses demographic perspectives as a natural way of linking the human and environmental dimensions. In the space available, I discuss primarily research that informs development (as highlighted in the UN SDGs ^4^). My first aim is to highlight important (but often neglected) areas of quantitative research, historical and prospective, that can contribute significantly to research and policy analyses. Secondly, I want to encourage discussion of the realities and complexities of such central factors as population change or values and culture.

I start with a simple general framework to highlight how demography and productivity shape human well-being. I use that framework to discuss three sets of issues and the corresponding challenges to modeling: first, population prehistory and early human development and their implications for the future; second, the challenges of a framework that incorporates multiple distinct dimensions of human and environmental well-being and the use of such a framework to explore the meanings of sustainability; and, third, inequality as a phenomenon triggered by development in the short run, and perhaps even in the long run, and models that examine changing inequality and its consequences. I conclude with a few words about other important (sometimes all-important) factors—political, institutional, and cultural—that affect human and environmental well-being. Throughout, I draw on perspectives from many disciplines, including demography, sociology, and economics as well as ecology, evolution, and environmental science.

I emphasize that my use of the term ‘models’ is not restricted to mathematical models but includes other types of models, such as computer-based games for one or many players, or visually rich interactive displays.

## Growth, demography, consumption: the big questions

Early agricultural populations ^7, 8^ were (mainly) dependent on food and demography. In the simplest case, a single population has W workers each producing a quantity Y food calories per year. These calories have to feed N people (including children and old people who do not work), so human well-being depends on

Average energy per capita J = (Y W)/N.

**Figure 3.**
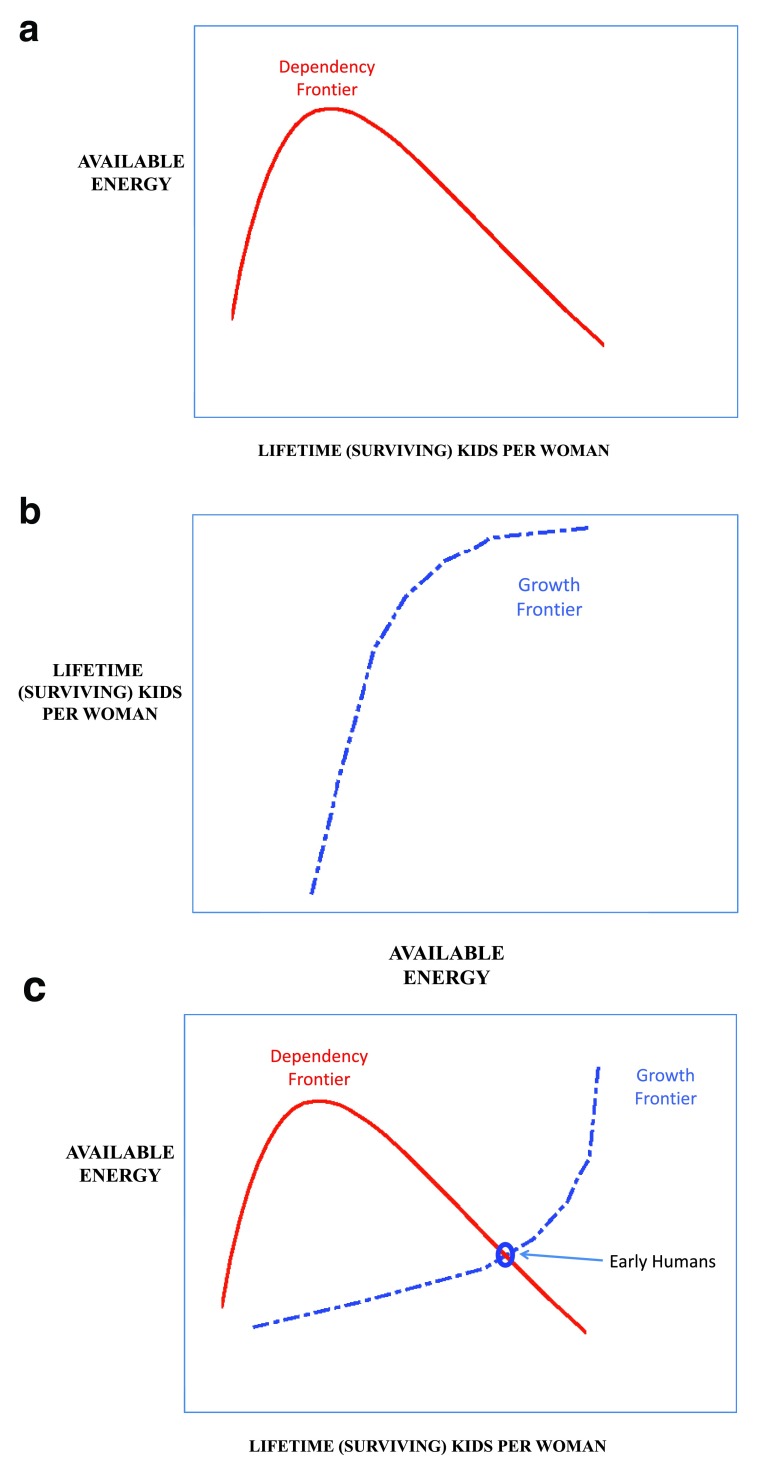
Prehistoric agriculture. ( **a**) The rate at which children are born increases with survival-weighted fertility. The proportion of workers rises with fertility but eventually falls as the proportion of children increases, forming a dependency frontier. ( **b**) Human fertility increases with available food energy but eventually saturates at some upper limit, forming a growth frontier. ( **c**) The intersection of the dependency and growth frontiers determines the prehistoric (Malthusian) equilibrium.

Here, age structure determines the ratio W/N of workers to total population. This ratio is low when fertility is low (and/or survival is high, implying more old people), peaks at intermediate fertility, and falls again when fertility is high (and/or survival is low, implying more young people); thus, we have a *dependency frontier* ( Figure 3a). Fertility and survival depend on available energy but saturate when energy available exceeds what can be physiologically used; thus, we have a *growth frontier* ( Figure 3b). For much of human history before about 1800, productivity Y changed slowly whereas fertility and survival could change fairly quickly; populations fluctuated around a stable, essentially Malthusian equilibrium (as shown in Figure 3c). But even in early history, this equilibrium was relevant only in some places and times; climate, migration, disease, and war often kept populations far from equilibrium, and a simplistic Malthusian picture rarely applies anywhere in the world today.

The Industrial Revolution led to a post-Malthusian world ^9^ in which average per-capita food energy ceased to be a major determinant of human well-being and fertility. Human well-being now depends on

Average consumption per capita J = (Y W)/N,

of an increasingly diverse set of consumables. The proportional growth rate of J is

r _J_ = (1/J)(dJ/dt) and

r
_J_ = r
_Y _+ r
_W_ – r
_N_ (Equation 1).

Over much of the 20th century, growth, measured by the rate of increase r _J_ of average per-capita consumption, became a principal measure of development. The relationship ( Equation 1) describes the historical experience of the rich countries over many decades: population structure (W/N) changed slowly and development (r _J _> 0) was largely due to growth in productivity Y (driven by technology). In recent decades, in the rich countries, populations are not growing, so r _N_ is near zero or may even be negative. The labor supply is static or shrinking as more people age out of the labor force than enter it, so r _W_ is also near zero or slightly negative. Thus, in the 21st century, we expect rich countries to grow at or below the rate of growth r _Y_ of productivity.

Equation 1 also describes the more recent experience of emerging economies such as China and India. In those countries, in the past two decades, population growth slowed (so r _N_ was small) but the labor force grew rapidly (so r _W_ >> r _N_ and was large, so r _J_ was large) and these economies grew rapidly. But India and China have diverged. Over the next two decades, India’s demographics will be much as before, but China’s labor force is shrinking (so for China, r _W_ < 0). It is not surprising that, in recent years, China’s net economic growth rate (the r _W_ in Equation 1) is falling and no longer benefits from demographic change.

### Key issues

This simple post-Malthusian view, of course, ignores (a) positive effects of population N on technology and thus on productivity Y, (b) negative effects of growth on local or global environment, (c) changes in the dimensions and measures of human well-being, (d) increases in the nature and level of consumption needed to maintain human well-being, and (e) the re-emergence of demographic constraints via the dependency ratio (W/N) as fertility declined and survival rates increased. These are the questions that models and policy aim to confront.

## Synthetic models of historical change

An essential aspect of a post-Malthusian world is the positive effect of population N on technological change, first established by Ester Boserup ^10^. Lee ^11^ formulated the first dynamic model that incorporates population growth, technological change, and their negative (Malthusian) and positive (Boserupian) feedbacks. An important feature of this model (and of the real world) is that changes in the level of technology determine multiple equilibria. Such changes in technology may be continuous or discontinuous. This model stimulated more sophisticated models of early agriculture ^8, 12^ and has been developed in the context of early human development and evolution ^13, 14^.

The structure of these demographically rich models can and should be extended to study historical population change, both in prehistory and in the past few centuries. Such models are also worth exploring in abstract and general ways to develop insights into the dynamics of human evolution, sustainability, persistence, and similar (slippery and complex) concepts. Particular examples of this are the following:

(a) Extending the models of Lee *et al*. ^12^ and Kirch *et al*. ^8^ to study the stability and sustainability of hunter-gatherer populations, the hunting-agriculture transition, and speeds of human migrations in pre-history and their effects on natural resources.

(b) Mapping Lee’s ^11^ multiple equilibria onto developmental paths past and present and the analysis of evolutionary versions of the model that capture the long-run transitions between equilibrium states.

(c) Exploring the relationship between Lee’s ^11^ model and Cohen’s (see Appendix 6 in ^15^) toy models of population-resource dynamics with time-dependent rates of renewal and exploitation. I note in passing that Cohen’s book has useful critiques of popular arguments (e.g. variants of Liebig’s rule and the notion of carrying capacity) that should be required reading for all environmentalists.

(d) The models discussed above and later in this article are built on the relationship between demography, resources, and technology. How do these models compare with simpler aggregate models (e.g. the model used by Turchin *et al*. ^16^)?

## The meaning of sustainable development

Sustainability is a term encompassing complex dimensions and processes and is hard to define operationally; see the discussion around the SDGs ^4^. Sustainable development is viewed by economists (at least those who have worked on human-environment linkages) as an equilibrium growth path (i.e. r _J _> 0 in Equation 1) that includes human and environmental well-being ^2, 17, 18^.

A central challenge is valuation: how to measure development in ways that incorporate multiple dimensions of both human well-being (see the indexes and reports accessible at http://hdr.undp.org/en) and environmental well-being. On the human side, data and models must at least describe human capital, socioeconomic condition, human health, the use of ecosystems and other natural resources, and local and global effects on the environment. On the environmental side, we must value a wider range of ecosystem services and describe ecosystem dynamics. And these human and ecosystem models have to be coupled.

The incorporation of such diverse elements of well-being is essential. The UN effort at measuring development in an integrated way ^2^ makes notable and important progress on combining human and ecosystem valuation but leaves out the value of human longevity. This is probably a fatal omission, given the priorities of people, especially the rich (e.g. the US National Institutes of Health spend over 4.5 times as much on health as the US National Science Foundation does on all other science). The development of measures of ecosystem services has greatly strengthened the assessment of environmental well-being (see, for example, the Natural Capital project, http://www.naturalcapitalproject.org/what-is-natural-capital/), but many problems remain ^19^.

Here is a short list of important open problems:

(a) Quantitative models that incorporate the direct and indirect consumption of renewable and non-renewable resources—one approach is to extend bioeconomic models for fisheries ^20^.

(b) Quantitative models that confront difficult trade-offs. For example, in India, there are about 2200 tigers and there are also about 700 million people who live on less than $2 US per day; how do we value investments in tiger conservation versus industrial development? We should explore models and methods that have been developed to assess multiple and poorly defined objectives such as fuzzy logic ^21^ or grade-of-membership ^22^.

(c) Models to quantify intergenerational effects for both humans and the environment. Intergenerational accounting was developed by economists ^23^ but has been applied only to pensions and taxation, as far as I know. Intergenerational effects clearly matter for the environment, but there has been only limited effort ^24^ to extend economic analysis to environmental well-being.

(d) Human aging worldwide is producing rapid change in the W/N ratio and has drawn enormous interest and analysis by demographers and economists (for just a taste, see Auerbach and Lee ^25^). Aging has been studied in the context of energy and carbon dioxide ^26– 28^ but not (as far as I know) in the broader context of effects on environments and sustainability.

(e) Life cycle transfers (from old to young and vice versa, both direct and mediated by taxation) are being studied globally in economic terms in the National Transfer Accounts project (see http://www.ntaccounts.org/web/nta/show/), and data are being collected and analyzed to study how “population growth and changing age structure influence economic growth, gender and generational equity, public finances …”. Extensions of this study are needed to incorporate the life cycle transmission of values/attitudes/ownership of ecosystems/services. These subjects appear to be little studied, and data from different countries can be usefully integrated into better models and better decisions.

## Inequality: an unexplored frontier

A temporary increase in economic inequality has long been expected to accompany development ^29^; in the longer term, inequality was supposed to shrink as economies became developed (i.e. rich). But a surprising and important corollary of rapid economic development is a large rise in spatial inequality in many aspects of human and environmental well-being.

Thus, in human health, Ram *et al*. ^30^ document large spatial differences in adult mortality by district in India, whereas Kumar *et al*. ^31^ find similar variation in mortality in children under age 5. These spatial differences within India are easily as large as average differences between India and, say, the US. Similar spatial inequality has been documented in China and other developing economies. Rapid development also generates growing economic inequality: Xie and Zhou ^32^ show, for example, that wage income inequality has been and is still rising rapidly in China. Similar trends have been found in India and other developing economies. There is also a rise in social inequality (e.g. in sex bias and the resulting imbalance in the marriage market). Thus, for China in recent years, Jin *et al.* ^33^ document a dramatic imbalance in the sex ratio of rural youth (what has become known as the problem of ‘Bare Branches’). In regard to environmental well-being, it is well known that there is spatial variation at many spatial scales in ecosystem services ^34^ (e.g. between rural and urban landscapes and ecosystems).

Kuznets notwithstanding, inequalities may persist and even widen in the long run. Piketty ^35^ has documented a recent and large rise in within-rich-country economic inequality; this is a surprise (unwelcome to many but perhaps not to everyone). Inequalities are likely to affect peoples’ preferences and willingness to pay for such things as environmental well-being.

We need analyses of the rise and consequences of spatial inequality that accompanies development. In particular, we need to examine (a) correlations between economic activity, migration, and human well-being; (b) path dependence as a driver of inequality in economics, demands on ecosystems, and environmental vulnerability (see Henning *et al*. ^36^ for an economic perspective); and (c) probabilistic methods to explore the performance of portfolios of ecosystems distributed in multiple ways: in physical space, in biological space (species, food webs, biomes, and so on), and in patterns of human resource use.

## Other models, agency, and institutions

Many elements of human-environment interactions are qualitative and may not be quantified easily or at all. Among these are the following: (a) cultural, ethical, moral, and religious differences in world views and individual decisions; (b) the uneven distribution of human agency and democracy (see Sen ^5^ for what I mean by the term ‘agency’); (c) stability or instability of governance and institutions; and (d) the potentially catastrophic effects of war, disease, or famine (these rarely follow a Malthusian script: see Sen ^5^, cited above, or accounts of the 2016 displacement of people from Syria).

Such qualitative factors mean that the study of human-environment interactions is ripe for exploration using new kinds of models, such as games and virtual reality simulations. I am not talking here about educational tools or about models that reinforce a particular (say, national) perspective; rather, we need tools that engage users from, and expose users to, diverse viewpoints and interests (economic, political, cultural, and institutional). The interactive and educational presentation of human-environment well-being can also exploit new tools to integrate and visualize databases (e.g. Google’s Fusion Tables, https://sites.google.com/site/fusiontableslab/home).

Scientists working on human-environment issues and development need to strengthen their engagement with institutions, especially those that shape key changes and attitudes (such as newspapers, media, and multinational institutions and corporations) across the world. For modelers (and others), a central aspect of this recognition is more, better, and targeted communication. But I stress that a goal of such communication is to engage, not preach.

## References

[1] Gerland P Raftery AE Sevčíková H: World population stabilization unlikely this century. *Science.* 2014;346(6206):234–7. 10.1126/science.1257469 25301627 PMC4230924

[2] UNU-IHDP and UNEP: Inclusive Wealth Report 2014. Measuring progress towards sustainability.Cambridge: Cambridge University Press,2014 Reference Source

[3] Wood SLR DeClerck F: Ecosystems and human well-being in the Sustainable Development Goals. *Front Ecol Environ.* 2015;13(3):123 10.1890/1540-9295-13.3.123

[4] United Nations Transforming our world: the 2030 agenda for sustainable development.General assembly Resolution A/RES/70/1,2015 Reference Source

[5] Sen A: Development as Freedom.New York: Knopf.1999 Reference Source

[6] Francis P: Address Of The Holy Father.South Lawn of the White House, Washington, D.C.,2015 Reference Source

[7] Lee CT Tuljapurkar S: Population and prehistory I: Food-dependent population growth in constant environments. *Theor Popul Biol.* 2008;73(4):473–82. 10.1016/j.tpb.2008.03.001 18439637

[8] Kirch PV Asner G Chadwick OA: Building and testing models of long-term agricultural intensification and population dynamics: A case study from the Leeward Kohala Field System, Hawai’i. *Ecol Model.* 2012;241:54–64. 10.1016/j.ecolmodel.2012.06.027

[9] Weil D: Economic Growth.Prentice Hall, 2nd edition.2008 Reference Source

[10] Boserup E: The condition of agricultural growth. The Economics of Agrarian Change under Population Pressure.Allan and Urwin, London,1965 Reference Source

[11] Lee R: Malthus and Boserup: A dynamic synthesis.In David Coleman and Roger Schofield (Eds.), *The State of Population Theory: Forward from Malthus* Oxford: Blackwell,1986; 96–130.

[12] Lee CT Puleston CO Tuljapurkar S: Population and prehistory III: food-dependent demography in variable environments. *Theor Popul Biol.* 2009;76(3):179–88. 10.1016/j.tpb.2009.06.003 19540865

[13] Richerson PJ Boyd R: Homage to Malthus, Ricardo, and Boserup: Toward a general theory of population, economic growth, environmental deterioration, wealth, and poverty. *Human Ecology Review.* 1998;4:85–90. Reference Source

[14] Wood JW: A Theory of Preindustrial Population Dynamics Demography, Economy, and Well‐Being in Malthusian Systems1. *Curr Anthropol.* 1998;39(1):99–135. 10.1086/204700

[15] Cohen JE: How Many people Can the Earth Support.Norton, New York,1995 Reference Source

[16] Turchin P Currie TE Turner EA: War, space, and the evolution of Old World complex societies. *Proc Natl Acad Sci U S A.* 2013;110(41):16384–9. 10.1073/pnas.1308825110 24062433 PMC3799307

[17] Dasgupta P: Nature in Economics. *Environ Resource Econ.* 2008;39(1):1–7. 10.1007/s10640-007-9178-4

[18] Parks S Gowdy J: What have economists learned about valuing nature?: A review essay. *Ecosystem Services.* 2013;3:e1–e10. 10.1016/j.ecoser.2012.12.002

[19] Guerry AD Polasky S Lubchenco J: Natural capital and ecosystem services informing decisions: From promise to practice. *Proc Natl Acad Sci U S A.* 2015;112(24):7348–55. 10.1073/pnas.1503751112 26082539 PMC4475956

[20] Kroetz K Sanchirico JN: The Bioeconomics of Spatial-Dynamic Systems in Natural Resource Management. *Annu Rev Resour Econ.* 2015;7:189–207. 10.1146/annurev-resource-083110-120047

[21] Herrera-Viedma E: Fuzzy sets and fuzzy logic in multi-criteria decision making. The 50th anniversary of Prof. Lotfi Zadeh's theory: Introduction. *Technological and Economic Development of Economy.* 2015;21(5):677–83. 10.3846/20294913.2015.1084956

[22] Airoldi EM Blei D Erosheva EA: Handbook of Mixed Membership Models and Their Applications.Boca Raton: CRC Press,2014 Reference Source

[23] Auerbach AJ Gokhale J Kotlikoff LJ: Generational Accounts - A Meaningful Alternative to Deficit Accounting.Cambridge, MA: National Bureau of Economic Research,1991 10.3386/w3589

[24] Rangel A: Forward and Backward Intergenerational Goods: Why Is Social Security Good for the Environment? *Am Econ Rev.* 2003;93(3):813–34. 10.1257/000282803322157106

[25] Auerbach AJ Lee RD, editors: Demographic Change and Fiscal Policy.Cambridge University Press, Cambridge,2008 Reference Source

[26] Liu J Daily GC Ehrlich PR: Effects of household dynamics on resource consumption and biodiversity. *Nature.* 2003;421(6922):530–3. 10.1038/nature01359 12540852

[27] O'Neill BC Dalton M Fuchs R: Global demographic trends and future carbon emissions. *Proc Natl Acad Sci U S A.* 2010;107(41):17521–6. 10.1073/pnas.1004581107 20937861 PMC2955139

[28] Zagheni E: The leverage of demographic dynamics on carbon dioxide emissions: does age structure matter? *Demography.* 2011;48(1):371–99. 10.1007/s13524-010-0004-1 21328039 PMC3059757

[29] Kuznets S: Economic Growth and Income Inequality. *American Economic Review.* 1955;45;1–28. Reference Source

[30] Ram U Jha P Gerland P: Age-specific and sex-specific adult mortality risk in India in 2014: Analysis of 0·27 million nationally surveyed deaths and demographic estimates from 597 districts. *Lancet Glob Health.* 2015;3(12):e767–e775. 10.1016/S2214-109X(15)00091-1 26566748

[31] Kumar C Singh PK Rai RK: Under-five mortality in high focus states in India: a district level geospatial analysis. *PLoS One.* 2012;7(5):e37515. 10.1371/journal.pone.0037515 22629412 PMC3356406

[32] Xie Y Zhou X: Income inequality in today's China. *Proc Natl Acad Sci U S A.* 2014;111(19):6928–33. 10.1073/pnas.1403158111 24778237 PMC4024912

[33] Jin X Liu L Li Y: "Bare Branches" and the Marriage Market in Rural China: Preliminary Evidence from a village-level survey. *Chin Sociol Rev.* 2013;46(1):83–104. 10.2753/CSA2162-0555460104 26213641 PMC4512178

[34] Arkema KK Verutes GM Wood SA: Embedding ecosystem services in coastal planning leads to better outcomes for people and nature. *Proc Natl Acad Sci U S A.* 2015;112(24):7390–5. 10.1073/pnas.1406483112 26082545 PMC4475972

[35] Piketty T: Capital in the 21st century.Harvard University Press, Cambridge.2014 Reference Source

[36] Henning M Stam E Wenting R: Path Dependence Research in Regional Economic Development: Cacophony or Knowledge Accumulation? *Regional Studies.* 2013;47(8):1348–62. 10.1080/00343404.2012.750422

